# The onset and the development of cardiometabolic aging: an insight into the underlying mechanisms

**DOI:** 10.3389/fphar.2024.1447890

**Published:** 2024-09-26

**Authors:** Sulogna Sarkar, Vani S. Prasanna, Pamelika Das, Hiroshi Suzuki, Kazuya Fujihara, Satoru Kodama, Hirohito Sone, Remya Sreedhar, Ravichandiran Velayutham, Kenichi Watanabe, Somasundaram Arumugam

**Affiliations:** ^1^ Department of Pharmacology and Toxicology, National Institute of Pharmaceutical Education and Research (NIPER)-Kolkata, Kolkata, West Bengal, India; ^2^ Department of Hematology, Endocrinology and Metabolism, Niigata University Graduate School of Medical and Dental Sciences, Niigata, Japan; ^3^ School of Pharmacy, Sister Nivedita University, Kolkata, West Bengal, India; ^4^ Director, National Institute of Pharmaceutical Education and Research (NIPER)-Kolkata, Kolkata, West Bengal, India; ^5^ Department of Laboratory Medicine and Clinical Epidemiology for Prevention of Noncommunicable Diseases, Niigata University Graduate School of Medical and Dental Sciences, Niigata, Japan

**Keywords:** cardiometabolic risk, aging, inflammation, immune dysfunction, mitochondrial dysfunction, oxidative stress, gut microbiome dysbiosis, genetic and environmental contributors

## Abstract

Metabolic compromise is crucial in aggravating age-associated chronic inflammation, oxidative stress, mitochondrial damage, increased LDL and triglycerides, and elevated blood pressure. Excessive adiposity, hyperglycemia, and insulin resistance due to aging are associated with elevated levels of damaging free radicals, inducing a proinflammatory state and hampering immune cell activity, leading to a malfunctioning cardiometabolic condition. The age-associated oxidative load and redox imbalance are contributing factors for cardiometabolic morbidities via vascular remodelling and endothelial damage. Recent evidence has claimed the importance of gut microbiota in maintaining regular metabolic activity, which declines with chronological aging and cardiometabolic comorbidities. Genetic mutations, polymorphic changes, and environmental factors strongly correlate with increased vulnerability to aberrant cardiometabolic changes by affecting key physiological pathways. Numerous studies have reported a robust link between biological aging and cardiometabolic dysfunction. This review outlines the scientific evidence exploring potential mechanisms behind the onset and development of cardiovascular and metabolic issues, particularly exacerbated with aging.

## 1 Introduction

### 1.1 Aging population and metabolic diseases

Aging is a complex, naturally occurring process resulting in a deterioration in the cellular homeostasis and increased vulnerability to stressors (Santamaria-Garcia et al., 2023). The multifactorial interplay of genetics, epigenetics as well as external and lifestyle-associated factors contributes to biological aging (Li Z. et al., 2021). The gradually changing demographic shift presents an increase in the elderly population, currently 700 million people above 65 years of (Nations, 2019). A growing body of evidence reports that several metabolic diseases expedite the progression of aging in humans (Tang et al., 2013; Suastika et al., 2011; Butler et al., 2006).

### 1.2 Epidemiology

The prevalence of metabolic syndrome in older people was found to be 72.9% in Mexicans, 42.4% in Taiwanese, 33.8% in Chinese, and 38% in Italians based on epidemiological evidence (Ortiz-Rodríguez et al., 2017; Yen et al., 2015; Laudisio et al., 2013). In contrast, the percentage rose from 13% to 50% in Indians and 34%–50% in Americans as they aged (Krishnamoorthy et al., 2020; Li et al., 2023). Recent studies conducted report age-related metabolic and cardiovascular risk in older people, ranging from 30% to 50% in China, 63.02% in the United States, and 62.5% in Iran (Zhao et al., 2023; Liang et al., 2023; Saeedi et al., 2023; Zhao et al., 2023; Liang et al., 2023; Saeedi et al., 2023).

### 1.3 Metabolic syndrome and cardiovascular risk

The metabolic syndrome centers on an array of metabolic disturbances like elevation in blood glucose levels, increased cholesterol, triglyceride levels, blood pressure obesity-mediated increased body mass index (BMI), and waist circumference (Bruce and Byrne, 2009; Lindblad et al., 2001; Ortiz-Rodríguez et al., 2017; Zhang et al., 2024). These multitude of conditions account for a significant risk of cardiovascular events, thus highlighting the cardiovascular consequences of metabolic stress, broadly referred to as cardiometabolic syndrome (Booth et al., 2006; Guo, Moellering, and Garvey, 2014). According to the National Cholesterol Education Program Adult Treatment Panel III (NCEP ATP III), International Diabetes Federation (IDF), or the World Health Organization (WHO) guidelines, cardiometabolic syndrome manifests via the presence of central obesity, elevated triglycerides, decreased HDL cholesterol, elevated blood pressure, and elevated fasting glucose levels (Liu et al., 2016b; Rezaianzadeh, Namayandeh, and Sadr, 2012; Alberti, Zimmet, and Shaw, 2006). Insulin resistance is central to WHO criteria, with inflammatory markers like C-reactive protein (CRP) increasingly recognized as part of the syndrome’s profile (Liu et al., 2016b; Patwary et al., 2022). The metabolic syndrome associated with increased insulin levels as well as low-grade inflammation causes endothelial damage and atherosclerotic plaque development, and its rupture as well as coronary artery disease (Tylutka et al., 2023; Li et al., 2023; Gumede and Khathi, 2023). The interaction between biological aging and cardiometabolic disturbances is complex but complementary.

### 1.4 Risk factors and aging

**FIGURE 1:**
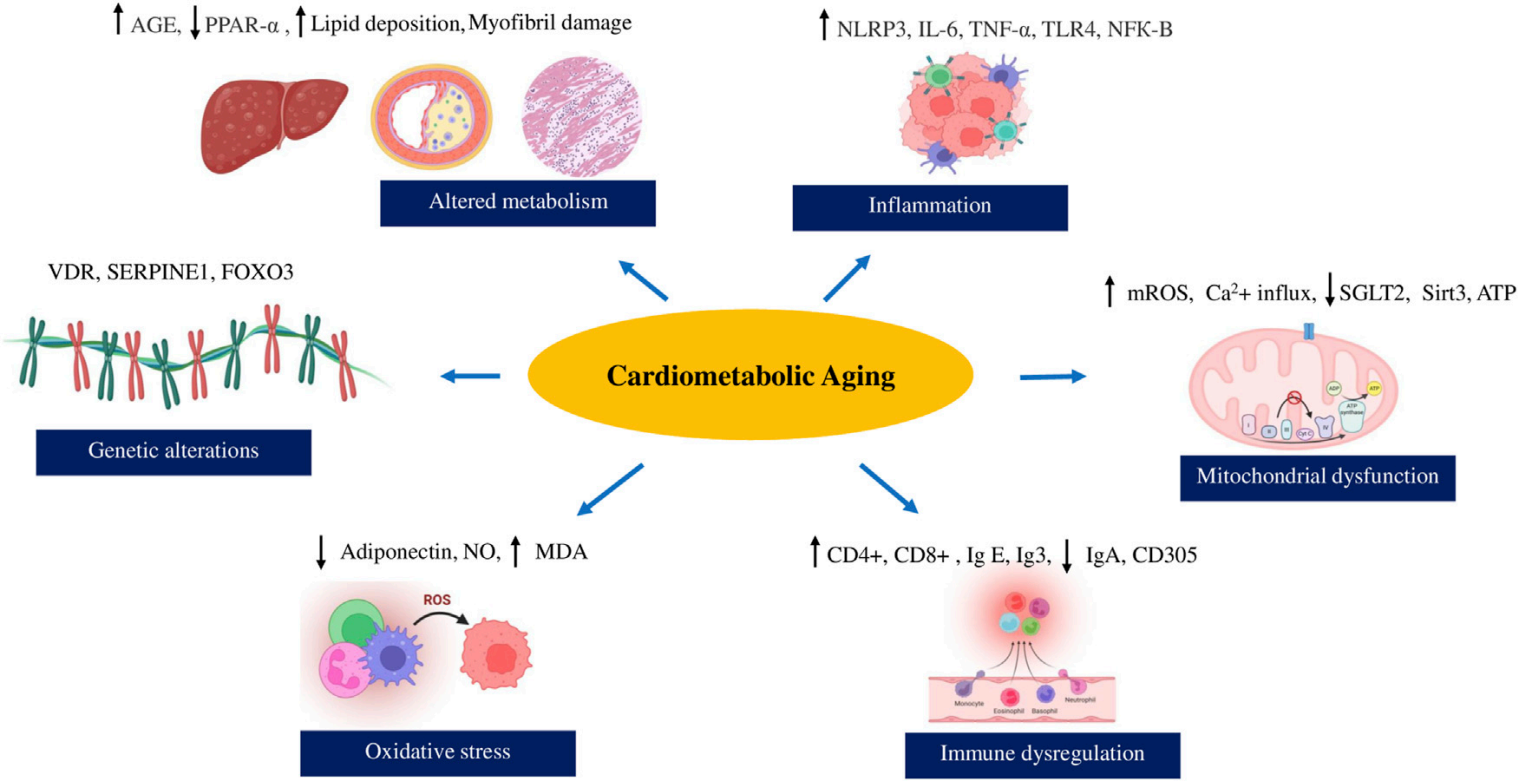
Major events linking cardiometabolic risk with biological aging.

According to literature reports, cardiometabolic syndrome’s severity and clinical manifestations increase with age, leading to an increased morbidity and mortality risk (Lindblad et al., 2001; Ortiz-Rodríguez et al., 2017). Metabolic abnormalities, cardiovascular health, and cellular aging strongly interact through a network of molecular changes and signaling cascades with age (Chimenti et al., 2003; Devrajani et al., 2023). Interestingly, the telomere length of aged human hearts with cardiac failure and the expression of senescent markers like p53, p21, and p16, 35, 36 exhibit similarities to those observed in metabolic syndrome associated with aging (Chimenti et al., 2003; Lee et al., 2022). Preliminary evidence suggests that reduction of metabolic activity is associated with increased obesity and cardiovascular events in the older and middle-aged population (Suastika et al., 2011; Butler et al., 2006; Gouveia et al., 2021). The presence of unhealthy lifestyles and habits like smoking also pave the way for aggravating cardiometabolic risk with age (Gouveia et al., 2021; Johari and Shahar, 2014). The senescence-associated inflammation and glucose imbalance disturb the immune signalling pathways and elevate cardiovascular risk through cytochrome nad-P21 mediated telomere uncapping in arteries of geriatric subjects (Zhang et al., 2021; Johari and Shahar, 2014; Morgan et al., 2013; Amano et al., 2019). Free radicals associated with mitochondrial dysfunction, changes in the gut microbiota, and epigenetic modifications adversely affect the cardiometabolic profile with aging in humans (Kim et al., 2018; Acharya and Talahalli, 2019; Bo-Htay et al., 2020; Biagi et al., 2010). Hence, we will explore the diverse mechanisms underlying cardiometabolic syndrome in the context of aging integrating epidemiological data, and incorporating molecular perspectives and clinical evidence. The major events linking cardiometabolic risk with biological aging is illustrated in Figure1. The clinical evidence of cardiometabolic risk in the elderly population has been enlisted in Table 1.

**TABLE 1:** The table enlists the clinical evidence of cardiometabolic risk in aging population.

Etiological factors	Clinical evidence based on aged population
Altered metabolism	(Ortiz-Rodríguez et al., 2017; Zhang et al., 2024; Tylutka et al., 2023; Gouveia et al., 2021; Suastika et al., 2011; Zha et al., 2017; Kizer et al., 2014; Finucane et al., 2014; Gradinaru et al., 2018; Wohlfahrt et al., 2014; Jung et al., 2022)
Inflammation	(Acharya and Talahalli, 2019; Xie et al., 2019; He et al., 2018; Baker et al., 2011; Jung et al., 2022; Marsland et al., 2010; Reynaert et al., 2016; Akbaraly et al., 2013; Zhang et al., 2023b; Zannas et al., 2019; Xin et al., 2019; Youm et al., 2012; Skoblow and Proulx, 2022; Al-Daghri et al., 2021; Giacconi et al., 2005)
Immune dysfunction	(Richard et al., 2017; Desdín-Micó et al., 2020; Bakun et al., 2014; Wang et al., 2017)
Mitochondrial dysfunction	(Niemann et al., 2011; Inal et al., 2001; Csiszar et al., 2002; Petersen et al., 2003; Fabbri et al., 2016; Tyrrell et al., 2020)
Oxidative stress	(Demissie et al., 2006; Karaouzene et al., 2011; Gradinaru et al., 2017; Bellanti et al., 2018; Maris and Ghitea, 2023; Sołtysik et al., 2022)
Gut microbiome dysbiosis	(Kahmann et al., 2008; Kamo et al., 2017; Afolayan et al., 2020; Pasini et al., 2016; Sandek et al., 2007; Amamoto et al., 2021; Li et al., 2019)
Genetic alterations	(Karuppagounder et al., 2017; Hua et al., 2015; Issa et al., 2016; Khan et al., 2017; Chen et al., 2020; Seidelmann et al., 2018; Yan et al., 2007; Sreedhar et al., 2016)
External and Lifestyle associated risk factors	(Ramírez-Manent et al., 2022b; Maas et al., 2020; Warrington et al., 2019; Artegoitia et al., 2021)

## 2 The major etiological factors

### 2.1 Altered metabolism

A metabolic shift during aging is characterized by reduced reliance on fatty acids as the primary energy source and increased glucose consumption, a hallmark feature of cardiometabolic aging in both humans and rodents (Liu et al., 2003; Suehiro et al., 2016; Zha et al., 2017; Moreau et al., 2004; Kizer et al., 2014). Senescence results in poor glucose homeostasis, insulin sensitivity, plasma adiponectin, and hemoglobin (HbA1c) levels (Hyyti et al., 2010; Finucane et al., 2014). The dysregulated adipokines, like low adiponectin and high leptin levels, also account for age-associated insulin and leptin resistance (Mohamad et al., 2014; Schautz et al., 2012). The age-associated impaired Wnt signalling contributes to vascular thickening and fibrosis (Fujimaki et al., 2015; Hu et al., 2020). The diminished estrogen levels play a salutary role in age-associated fat accumulation and insulin resistance through alternative Wnt signaling pathways due to an imbalance in the Wnt5A and SFRP5 in aged females (Marmentini et al., 2021; Fayaz et al., 2019). The cumulative effects of lipid accumulation associated with insulin resistance in elderly individuals aggravates coronary artery necrosis (DeNino et al., 2001; Lee et al., 2009). A significant decline in the insulin growth factor (IGF) levels mediates cardiac fibrotic pathway activation via the Akt/Rho kinase 2/α-smooth muscle actin cascade (Bian et al., 2020). The age-associated decline in the AMPK and PGC-1α/PPAR-α signaling, correlates with increased rodent adiposity (Zang et al., 2004; Reznick et al., 2007; Alberti, Zimmet, and Shaw, 2006). Aged myocardium in monkeys responds to metabolic stress induced by high-fat sugar diet via increased infiltration of lymphocytes, cytokines, and lipids, leading to poor actomyosin MgATPase activity and altered myofilament phosphorylation (Zheng et al., 2021).

An upsurge in the release of alanine transferase (ALT), aspartate transferase (AST), urea, creatinine, and calcium exacerbate metabolic stress with aging (Liu et al., 2016b; Kim et al., 2010). The ABCA1 pathway, linked to reverse cholesterol transport (RCT) via high-density lipoproteins (HDL), diminishes with aging, along with changes in the HDL composition and phospholipidic bilayer fluidity (Berrougui et al., 2007). High lipid induces upregulation of clusterin and alanine transferases, leading to steatosis as well as elevated osteopontin levels, contributing to cardiac fibrosis and myocardial aging (Liu et al., 2016b; Bradley et al., 2019; Sawaki et al., 2018). The reduced levels of adipokines like leptin and adiponectin with aging disrupt metabolic regulation and affect cardiovascular function (Mohamad et al., 2014; Gradinaru et al., 2018).

Aging aggravates atherosclerotic plaque accumulation by increasing low-density lipoprotein (LDL) and cholesterol in the aorta, potentially driven by the overexpression of liver cholesterol protein transporters (ABCG5 and ABCG8) and the migration of monocytes and macrophages into adipose tissues (Palmer et al., 2019; Simo et al., 2021; Malgor et al., 2014). The senescent cells mediated adipose tissue hypertrophy contribute to left ventricular stiffness and poor left ventricular ejection fraction (Djoussé et al., 2012; Wohlfahrt et al., 2014). This may result in heart failure with preserved ejection fraction (HFpEF) and coronary artery disease during aging (Jung et al., 2022; Canepa et al., 2012; Prineas, Folsom, and Kaye, 1993).

### 2.2 Inflammation

The telomere dysfunction and DNA damage during aging contributes to inflammation via p53 activation which in turn, promotes the downregulation of peroxisome proliferator-activated receptor gamma coactivator one alpha (PGC-1α) and mitochondrial Sirts (Sirt3/4/5) (Li N. et al., 2021; He et al., 2017). Aging raises the glycemic index and lipid levels and upsurges inflammatory mediators and free radicals (Acharya and Talahalli, 2019; Aquino-Martinez et al., 2021; Wang et al., 2016). Aging upregulates the TLR4 expression in the aorta, raising the serum lipoprotein levels and affecting insulin sensitivity, cardiac function, and vascular relaxation (Xie et al., 2019; Malgor et al., 2014). The TLR4 via AGE causes inflammation, leading to the macrophage transitioning to a pro-inflammatory state, reduced insulin sensitivity, and pancreatic beta cell damage with age (Liu et al., 2021a; He et al., 2018). The senescence-associated secretory phenotype (SASP) manifests via RAGE (Receptor for Advanced glycation end products) cascade modulation, leading to the release of IL-6, IL-1β, CRP, and TNF-α release in the fatty tissues and renal tubular cells (Liu et al., 2014; Baker et al., 2011). The upregulated TNF-α levels increase insulin resistivity and inhibit the insulin receptor phosphorylation and atheromatous plaque deposition with aging (Nilsson et al., 1998; Skoog et al., 2002). Increased IL-6 levels, linked with insulin resistance, have been associated with cardiometabolic risk in older and middle-aged people (Marsland et al., 2010; Onder et al., 2002).

Cellular aging evokes an inflammatory response within the vascular wall via perivascular lipid accumulation and endothelial insult in the heart (Akbaraly et al., 2013; Reynaert et al., 2016). Increased expression of FKBP5 and NFκB drives cellular aging and may account for cardiovascular inflammation with age (Zannas et al., 2019; Zhang et al., 2023a; Sabbagh et al., 2014; Rovillain et al., 2011). The decrease in the catecholamine-mediated lipolysis with age is due to the reduction in the noradrenaline bioavailability via adipose tissue macrophages through upregulation of the NLRP3 inflammasome and MAO-A with growth differentiation factor-3 (GDF3) playing a significant role (Camell et al., 2017). Vascular aging corresponds with a significant increase in free radicals and inflammatory mediators such as IL-6 and IL-1β in obese rodents, which can be improved through therapies targeting senescent cells (Xin et al., 2019; Succurro et al., 2008). Higher IL-6 expression contributes to diabetes and atherosclerosis, especially in elderly patients (Giacconi et al., 2005). The build-up of plaques with age, caused by the activation of the NLRP3 inflammasome by IL-1β, may lead to cholesterol aggregation and disrupted insulin levels, consequently promoting further IL-1β release (Youm et al., 2012; Butcher et al., 2014). The mitochondrial H_2_O_2_ mediated TNF-α, ICAM, and iNOS levels potentially enhance the NF-KB activity in the arteries of the aged rats; moreover, inhibition of the TNF-α cascade could ameliorate the early senescence via immunometabolic regulation (Ungvari et al., 1999; Desdín-Micó et al., 2020). The high C-reactive protein (CRP) associated inflammation is linked to enhanced telomerase activity, leading to telomere shortening and diabetes-related premature senescence, and accelerated aging in older adults (Skoblow and Proulx, 2022; Desdín-Micó et al., 2020; Al-Daghri et al., 2021).

### 2.3 Immune dysfunction

The compromised immune response is associated with an upsurge in the release of inflammatory mediators and disrupted glucose homeostasis in aged obese rats (K. Kim et al., 2018). In response to aging, immune cells like macrophages exhibit an excess release of CD4^+^ and CD8^+^ T cells, reactive oxygen species (ROS), and increased senescent accumulation in the fatty tissues of old mice (W. He et al., 2018; Lumeng et al., 2011). An upregulation of immune-related genes with age includes chemokines (CCLs 6, 8, 9), complement proteins (C1qa, C1qb, C1qc, C3, C4b), lysozymes, and pro-inflammatory caspases (Casp1, Casp4, Casp12) in the ventricles of aged mice (Bartling et al., 2019). The immunosenescence likely occurs via the sestrin-mediated MAPK signaling, which, in turn, results in age-driven thymus degeneration and lowered T cell count in obese mice (Gress and Deeks, 2009; Lanna et al., 2017). Compromised T-lymphocyte and neutrophil activity precipitates metabolic syndrome in humans, with mitochondrial transcription factor-A deficiency causing cardiometabolic inflammation and neutrophil oxidative stress promoting atherosclerosis and senescence in mice (Richard et al., 2017; Desdín-Micó et al., 2020).

The increased mast cell markers like immunoglobulin-E (IgE), chymase, and tryptase lead to heightened inflammatory response and metabolic disturbances with aging (Wang et al., 2017). In human and zebrafish models, aging facilitates complement activation of the innate response via elevated Ig3 and decreased IgA and CD305 levels (Bakun et al., 2014; Reuter et al., 2022). The immunosenescence-related cardiometabolic complications include an upsurge in the natural killer, and T cells mediated IFN-γ release from the adipose tissues as well as high levels of oxidative stress in aged rodents (Srivastava et al., 2016; Palaniyappan and Alphonse, 2011). The increased infiltration of macrophages and T cells mediates fibrotic remodelling associated with systolic and diastolic dysfunction in aged mice hearts (Martini et al., 2020). The aging exacerbated immunoregulatory changes are primarily associated with inflammatory responses and metabolic disturbances, with implications for cardiometabolic health in both humans and animal models.

### 2.4 Mitochondrial dysfunction

Senescence enforces autophagy and mitochondrial fission via Drp1, PINK1, and laminin on mitochondria, expediting cardiometabolic damage in rodents (Duan et al., 2020; Madrigal-Matute, Cuervo, and Sluimer, 2022; Zha et al., 2017). The impaired FASL/FAS/TNF-α pathway, a major stimulus in age-associated pathologies, activates the caspase-associated mitochondrial apoptosis with age (Lai et al., 2014; Lagunas-Rangel, 2023). A rise in glycolysis in aging mouse hearts is related to a decline in the mitochondrial oxidative phosphorylation (OXPHOS) genes, affecting mitochondrial function (Serio et al., 2023). Aging increases mitochondrial calcium permeation compromises SGLT2 function and alters mitochondrial permeability and potential, consequently limiting cardiac function (Endlicher et al., 2021; Olgar et al., 2020). At the cellular level, these changes in mitochondria are accompanied by an increase of ROS and disturbed balance of fusion-fission (such as Drp1/MFN1/2) leading to age-related cardiac aging via autophagy/mitochondrial fission/pro-apoptotic signaling pathways (Bo-Htay et al., 2020; Niemann et al., 2011). The reduced oxidative phosphorylation and state III mitochondrial respiration were reported in the mitochondria of elderly patients with insulin resistance and high intramyocellular and liver triglyceride levels, as reported via 13 C NMR and 31 P NMR studies (Petersen et al., 2003; Fabbri et al., 2017). Aged rats experience decreased electron transport chain (ETC.) activity, reduced NADH dehydrogenase and cytochrome C oxidase, and cardiolipin-associated free radical damage (Kumaran et al., 2004). This decline in, ETC., function and membrane potential can reduce mitochondrial copy number, a well characterized sign for loss of metabolic function (Dang et al., 2017; Liu et al., 2021b). In other rodent models of aging, the diminished myocardial fatty acid oxidation (MFAO) seems to be linked to an age-related decrease in carnitine palmitoyltransferase-1 activity, the rate-limiting enzyme for mitochondrial long-chain fatty acid uptake (Mcmillin et al., 1993; Gómez, Heath, and Hagen, 2012). Elevated levels of IL-6, ATG5, and Parkin underscore the importance of autophagy and mitophagy in atherosclerosis, independent of lipid accumulation (Tyrrell et al., 2020).

Aging manifests a significant decrease in the mitochondrial enzymes like succinate dehydrogenase and superoxide dismutase, which correlates to reduced left ventricular function and sympathetic vagal activity in diabetic and atherosclerotic patients (Inal et al., 2001; Csiszar et al., 2002; Singh et al., 2015; Fetterman et al., 2016; Balietti et al., 2010). The concurrent rise in the mitochondrial hydrogen peroxide (H_2_O_2)_ levels in the arteries of aged rats may result from a substantial increase in mitochondrial superoxide (O2•−) levels, with manganese superoxide dismutase (MnSOD) aiding its conversion (Csiszar et al., 2002; Han, Williams, and Cadenas, 2001). The mitochondrial H_2_O_2_ triggers NF-κB via a reinforced inflammatory response during vascular aging, resulting in insulin resistance in both aged rats and humans (Ungvari et al., 2007; Niemann et al., 2011). The endothelial superoxide levels increase exorbitantly with age due to the hyperacetylation of mitochondrial superoxide dismutase (SOD), which, in turn, raises the blood pressure, possibly via reduced Sirt3 expression in mice (Dikalova et al., 2017; X; He et al., 2017). Another hypothesis is that upregulation in the Sirt3 levels may result from a compensatory mechanism against cardiovascular aging in mice (Brown et al., 2013).

### 2.5 Oxidative stress

Hypertensive conditions can raise oxidative stress and insulin resistance, culminating in a reduced free radical scavenging activity of antioxidants in both the heart and plasma and decreased leukocyte telomere length, a characteristic feature of aging (Demissie et al., 2006; Sivoňová et al., 2007). Age-related cardiovascular dysfunction stems from impaired antioxidant pathways, DJ-1, and FOXO transcriptional pathways, exacerbating atherosclerosis, while ActR II signaling linked cardiovascular aging to metabolic damage (Collins et al., 2009; Roh et al., 2019; R; Chen et al., 2020). Aging culminates in a marked decline in the Nrf2, FOXO, and PGC-1α associated pathways, resulting in poor antioxidant capacity via decreased gene expression (Gureev et al., 2022; Medoro et al., 2023; Du and Zheng, 2021; Birnbaum et al., 2019; Halling et al., 2019; Angulo et al., 2019). The diminished antioxidants like glutathione reductase, superoxide dismutase, catalase, and glutathione decrease (GSH), may contribute to a substantial increase in the malondialdehyde (MDA) levels in both aged rodents and humans (Ramesh et al., 2012; Karaouzene et al., 2011). Age-mediated visceral fat accumulation leads to oxidative injury via increased superoxide anion generation and lipid peroxides, which, in turn, results in higher glucose levels, intracellular adhesion molecule 1(ICAM-1) and Von Willebrand factor (vWFl) and LDL cholesterol (Sołtysik et al., 2022). In elderly patients, the obesity-associated loss of muscle mass is accompanied by a rise in the GSSG/GSH ratio and plasma MDA/HNE protein adducts, along with increased carotid intima-media thickness (IMT) (Bellanti et al., 2018; Maris and Ghitea, 2023). Lipid peroxidation in aged rodent hearts, attributed to modulated Na/K-ATPase ROS signaling, increases oxidative damage, reduces sulfhydryl groups, and causes DNA damage in lymphocytes (Sudheesh et al., 2010; Sivoňová et al., 2007). The dysregulated redox activity-mediated cardiometabolic damage decreases adiponectin levels with age in human subjects (Gradinaru et al., 2017).

Aging, accompanied by an increased Nox-4 and reduced nitric oxide (NO) expression levels in cardiomyocytes, may lead to poor arterial elasticity, endothelial vasodilation, and left ventricular damage in aging mice (Ago et al., 2010; Lesniewski et al., 2009). Age-related endothelial injury, through increased angiotensin I and II receptor expression, stimulates hydrogen peroxide and superoxide ion release in mesenteric arteries (Khodja et al., 2012). In obese mice, aging is linked with the fibrotic remodelling of the heart and oxidative damage to mitochondrial DNA, which is repressed by antioxidants like catalase (Dai et al., 2009). There is an association between aging and elevated levels of alanine transferase (ALT), aspartate transferase (AST), creatinine, and serum urea nitrogen in rats. At the same time, free radical damage and immune system potentiation are associated with age-dependent oxidative damage (Li et al., 2007). Thus, oxidative damage acts as a major impetus for age-related cardiometabolic pathologies and underscores the significance of antioxidant therapy in addressing age-related cardiometabolic complications.

### 2.6 Gut microbiome dysbiosis

The primary immunological modifications linked with aging are decreased T cell activity, increased inflammation, and IL-10, IgA, IgM, and IgG-associated poor gut metabolites activity (Zhang et al., 2021; Kahmann et al., 2008). The changes in gut microbe composition with aging alter the gut permeability and disrupt the immune defenses, inducing systemic inflammation and cardiovascular risk via increased endotoxin access into the bloodstream in elderly patients (Biagi et al., 2010; Anker et al., 1997). The deteriorating gut microbiome and dysregulated glucose and lipid levels exacerbate inflammation and immune T cell-mediated plaque deposition in the mucosal lining (Toubal et al., 2020). Age-related gut microbiome changes in heart failure subjects include decreased levels of Eubacterium rectale, Dorea longicatena, and Bacteriodetes and increased levels of *Lactobacillus* and Proteobacteria (Kamo et al., 2017). In elderly diabetic individuals, Actinobacteria and Corinobacteriaceae microbes significantly increase compared to those without diabetes (Afolayan et al., 2020). Increased *Candida*, *Campylobacter*, and *Shigella* levels are linked to high intestinal permeability, inflammatory response, as well as right atrial pressure, while increased *Firmicutes/Bacteroidetes* microbes’ ratio is associated with enhanced insulin sensitivity in aged humans and rodents (Liu et al., 2016a; Pasini et al., 2016). Probiotics and beneficial gut bacteria like *Bacteroides*, Eubacterium rectale, and *Fusobacterium* prausnitzii reduce the age-related rise in diastolic blood pressure, cardiac infarct size, and intestinal wall thickness in older heart failure patients (Amamoto et al., 2021; Sandek et al., 2007).

The intestinal healthy bacteria replenishment promotes enhanced insulin responsiveness with interleukin-37 therapy in aged diabetic patients (Li et al., 2019). Microbial metabolites of protocatechuic acid, enterolactone, and Fufang Zhenshu TiaoZhi (FTZ) confer enhanced sphingolipid and arachidonic acid metabolism for cardioprotective and anti-atherosclerotic benefits in both adult and aging mice respectively, showing promise as a potential therapy for mitigating cardiometabolic risk (Wang et al., 2012; Luo et al., 2020). The changes in the microbial composition could be linked to cardiovascular and metabolic pathologies or may represent a response to changes in the gut environment associated with cardiometabolic risk in older individuals.

### 2.7 Genetic alterations

Cellular aging, a stable cell-cycle arrest, is a crucial feature of aging and age-related diseases, characterized by the activation of cell-cycle regulators like p53/p21 WAF1/CIP1 and p16 INK4A (Lee et al., 2022). Age-related metabolic reprogramming of the genomic profile includes changes in the CDKN2B/CDKN2A, FHIT, TDRG1, and ELAVL4, which correlates to high glucose levels, insulin release, and insulin resistance (Hribal et al., 2011). The mutated caveolin gene reduces Cav1 expression, lowering HDL and insulin levels and decreasing the prevalence of metabolic syndrome (Baudrand et al., 2015). Meanwhile, the senescent-associated mouse prone-8 (SAMP-8) correlated with mitochondrial damage, ER stress, cytokine release, and cardiac fibrosis (Karuppagounder et al., 2017). In aged populations, the mutated vitamin D receptor (VDR) gene reduces the LDL and cholesterol levels, whereas the plasminogen activator inhibitor-1 encoding SERPINE1 gene alterations decrease senescence alongwith improved metabolic prifile (Issa et al., 2016; Khan et al., 2017). The FOXO3 gene variant lowered age-related blood pressure and glucose increase, while the SGLT variant raised blood glucose levels with aging (R. Chen et al., 2020; Seidelmann et al., 2018). The aging adrenomedullin-deficient mice showed increased oxidative stress linked to insulin resistance compared to the control mice (Shimosawa et al., 2003). In contrast, old adenyl cyclase 5-deficient mice reported higher levels of the antioxidant SOD in order to combat age-related cardiovascular pathologies (Yan et al., 2007).

The transcriptional coactivator p300/CBP promotes cardiovascular aging via enhanced histone marker Hk2 expression through H3K27ac deposition (Serio et al., 2023). The reduced cardiac contractile damage and increased calcium levels in mice lacking cathepsin decrease age-related biomarkers, including p21, p16, and cardiac lipofuscin (Hua et al., 2015). Klotho deficiency impacts the Nrf2-GR pathway, affecting Glutathione reductase expression and activity in the heart, leading to oxidative stress-mediated cardiac aging (K. Chen et al., 2021). The age-associated decrease in the ejection fraction and fractional shortening in the hearts of senescence-accelerated mouse prone 8 (SAMP-8) mice can be attributed to the p38 MAPK pathway and ER stress-induced apoptosis (Sreedhar et al., 2016). These changes coincide with alterations in the extracellular matrix, diminished cellular interactions between gap junctions and connexin-43, and increased matrix metalloproteinase-2 (MMP-2) levels (Nagibin et al., 2016).

### 2.8 External and lifestyle associated risk factors

Cardiometabolic remodeling through external manipulations has become increasingly evident with biological aging. External factors like inadequate macronutrient intake-induced metabolic damage and accelerated aging via mTOR activation aggravate cardiovascular risks (Solon-Biet et al., 2014; Schloesser et al., 2015). Reduced hepatic mTOR activity via dietary restriction leads to longevity via reduced Akt and S6 kinase (Schloesser et al., 2015). Consumption of a healthy diet with age lowered the predisposition of cardiometabolic risk (Artegoitia et al., 2021). The nutritional intake and health conditions of the mothers also play an essential role in predisposing offspring towards age-associated cardiometabolic impairments like vascular aging (Warrington et al., 2019; Mora-Urda, Acevedo, and Montero López, 2019). The age-related diastolic dysfunction and oxidative stress-mediated DNA damage were reportedly lowered via calorie restriction along with an improvement in the lifespan of mice models (Sohal et al., 1994; Taffet, Pham, and Hartley, 1997). The age-associated mitochondrial DNA accumulation in the mice’s hearts lowered with improved food intake (Melov et al., 1997). Aging correlates with increased expression of collagen III, α-SMA, TGF-β, Bax, SOD, NRF2, and HO-1 in mice hearts, as well as telomere stabilizing proteins TRF2, TERT, cardiac insulin-like growth factor (IGF), and e-NOS (Werner et al., 2008; Pei et al., 2021). Mild exercise significantly lowered these protein’s expression, ameliorating fibrotic remodeling, apoptosis, and oxidative injury with aging in mice (Werner et al., 2008; Pei et al., 2021). The increased vulnerability of elderly smokers towards developing cardiometabolic risk could be due to smoking-triggered DNA methylation and gene alterations (Maas et al., 2020; Ramírez Manent et al., 2022a). Thus, the diverse genetic mutations could be decisive for developing several clinical phenotypes of cardiometabolic conditions with the onset of aging.

## 3 Discussion

The complex nature of aging is becoming more apparent, highlighting its importance as a significant multifaceted risk factor for the onset of cardiometabolic diseases and *vice versa*. Recent evidence highlights the clinical value of diagnostic indicators such as Leukocyte alkaline phosphatase (LAP), TNF-α, and HMGB-1 in predicting metabolic syndrome characterized by increased lipid accumulation in aging.

Aging at the cellular level involves the SASP phenotype with increased p53 and p21, causing cell cycle arrest, resulting in G1 cell cycle arrest and p16-mediated S-phase arrest in the central metabolic organs. The metabolic insult raises the senescence-associated β-galactosidase (SA-β-gal) levels, decreases Beclin1 and LC3II, compromises autophagy, and induces caspase-3-mediated apoptosis in cardiomyocytes. The age-dependent increased p16 INK4a, telomerase, MCM5, and p53 diminish the cardiac cell regeneration. ER stress from misfolded proteins like Glucose regulated protein 78 (GRP78) and C/EBP Homologous protein (CHOP) activates the p38 MAPK cascade and promotes caspase-12-mediated apoptosis with age.

Increased liver adiposity results in low C-peptide-associated beta cell dysfunction, reducing glucose sensitivity and insulin extraction from the liver. Poor insulin clearance in aging is linked to Carcinoembryonic Antigen-Related Cell Adhesion Molecule 1 (CEACAM1) and insulin-degrading enzyme (IDE), affecting insulin metabolism. The diminished glucose and fat metabolism accounts for the changes in adipokines like adiponectin leptin, contributing to age-related insulin resistance. This resistance further aggravates inflammatory mediators (TNF-α, IL-6, and IL-1β), which induce serine phosphorylation of insulin receptor substrate-1 (IRS-1), disrupting insulin signaling by impairing the interctions among IRS-1, phosphatidylinositol 3-kinase (PI3K) and protein kinase B (Akt).

Aging-driven lipid-associated inflammation activates NF-κB and Janus kinase (JNK) pathways, while excess free fatty acids (FFAs) and lipid intermediates like diacylglycerol (DAG) affect insulin signaling via PKC activation. Chronic endoplasmic reticulum (ER) stress worsens insulin resistance via the unfolded protein response (UPR). The higher prevalence of age-related adiposity in older women than men can be due to the reduced estrogen level mediated reduction in the AMPK activity leading to reduced fatty acid oxidation and increased lipid accumulation. Lower estrogen also impairs eNOS activity and enhances TGF-β signaling, promoting endothelial damage and fibrosis.

The high CRP levels associated with inflammation can primarily act through NLRP3 inflammasome, PPAR signaling, and the HIF-1α pathway, affecting cardiometabolic homeostasis with age. The AGE-mediated ER stress results in an age-associated proinflammatory phenotype via glucose-regulated protein 78 (GRP78), cell-cycle regulator p21 and RAGE. The alteration of zinc homeostasis with age due to deficiency and malabsorption induces polymorphic changes, particularly in metallothionein, resulting in cytokine storms. Multiple clinical studies have consistently shown a strong relationship between aging and chronic inflammation, regardless of socioeconomic status, health behaviors (such as smoking and physical activity), or the use of anti-inflammatory medications. The activity of mitochondrial complex I, NADH oxidoreductase, declines significantly with age, with reduced protein carbonyl content and oxidative DNA damage (8-hydroxy-2′-deoxyguanosine) in cardiomyocytes and results in apoptosis with increased cytochrome-C, Bax, and Bcl-xS release.

Age-associated changes in the expression of Drp1, PINK1, and laminin drive autophagy and mitochondrial fission, while the impaired FASL/FAS/TNF-α affect the mitochondrial function, leading to increased ROS, disturbed fusion-fission balance, and reduced metabolic and cardiac efficiency. The significant age-related changes involve dysregulated DJ-1, FOXO-1, Na/K-ATPase ROS, and ActR II pathways, culminating in an increased oxidative injury which correlates with remarkably high levels of MDA, SOD, and Glutathione peroxidase (GPx) with age. Age-related changes in the gut microbial environment include reduced short-chain fatty acids (SCFAs), such as butyrate produced by Eubacterium rectale and Dorea longicatena which affect gut health. Age-related increase in gut permeability promotes the translocation of endotoxins and microbes in the systemic circulation, resulting in compromised immune defence and enhanced inflammatory response during aging. These alterations lead to insulin resistance and cardiovascular risks in the aging population. Increased mitochondrial H_2_O_2_ provokes inflammatory responses, promoting insulin resistance and hypertension, potentially involving mechanisms related to Sirt3 expression during aging. The impaired signalling cascades like DJ-1, FOXO, ActR II, Nrf2, and PGC-1α play a vital role in the downregulation of antioxidant enzymes like glutathione and superoxide dismutase in age-related cardiometabolic pathologies. Delving deeper into these pathways and target proteins could serve as biomarkers for assessing cardiometabolic health in aging populations and may offer promising avenues for therapeutic intervention. Future research could focus on developing diagnostic tools that measure these proteins’ activity or localization to predict the risk of several cardiometabolic events in older adults. Several studies have identified genetic background as a link between metabolic and cardiac remodelling with age, including gene-related changes like klotho, adrenomedullin, SAMP-8, cathepsin, VDR, and SERPINE1. These findings offer new models for studying age-related disorders, paving the way for gene-directed therapies.

In conclusion, the aging population presents a significant challenge for healthcare systems worldwide, necessitating a deeper understanding of the physiological changes and risk factors associated with the aging process. A comprehensive strategy addressing cardiometabolic aging should incorporate medical and nutraceutical therapies as well as lifestyle modifications, including a healthy diet, regular physical activity, and discontinued smoking habits. Additionally, gene profiling may significantly impact patient medication responses, highlighting the importance of personalized treatment approaches. Customizing these approaches to individual patient’s needs and consistently monitoring progress can enhance effectiveness and improve health outcomes for the elderly population. Collaborative efforts between healthcare providers, researchers, and policymakers are crucial in effectively addressing the challenges of the aging population.

## Abbreviations

Nrf2, Nuclear factor erythroid 2-related factor 2; FOXO, Forkhead box protein O1; PPARα, Peroxisome proliferator-activated receptor alpha; Drp1, Dynamin-related Protein; Mfn1, Mitofusin-1; NLRP3, NOD-like receptor family pyrin domain containing 3), NFКB, Nuclear factor kappa-light-chain-enhancer of activated B cells; PI3K, phosphoinositide 3-kinase; CD 4, Cluster of differentiation 4; IL-6, Interleukin-6; TNF-α, Tumor necrosis factor-α.
